# Novel Imaging Biomarkers for Huntington’s Disease and Other Hereditary Choreas

**DOI:** 10.1007/s11910-018-0890-y

**Published:** 2018-10-05

**Authors:** Patrik Fazio, Martin Paucar, Per Svenningsson, Andrea Varrone

**Affiliations:** 1 Department of Clinical Neuroscience, Centre for Psychiatry Research, Karolinska Institutet and Stockholm County Council, R5:02 Karolinska University Hospital, SE-171 76 Stockholm, Sweden; 2 Department of Neurology, Karolinska University Hospital, Stockholm, Sweden; 3 Department of Clinical Neuroscience, Centre for Molecular Medicine, Karolinska Institutet, Stockholm, Sweden

**Keywords:** Huntington’s disease, Huntington-like, Chorea, Neuroimaging, Pharmacodynamic biomarker, Diagnostic tool

## Abstract

**Purpose of the Review:**

Imaging biomarkers for neurodegenerative disorders are primarily developed with the goal to aid diagnosis, to monitor disease progression, and to assess the efficacy of disease-modifying therapies in support to clinical outcomes that may either show limited sensitivity or need extended time for their evaluation. This article will review the most recent concepts and findings in the field of neuroimaging applied to Huntington’s disease and Huntington-like syndromes. Emphasis will be given to the discussion of potential pharmacodynamic biomarkers for clinical trials in Huntington’s disease (HD) and of neuroimaging tools that can be used as diagnostic biomarkers in HD-like syndromes.

**Recent Findings:**

Several magnetic resonance (MR) and positron emission tomography (PET) molecular imaging tools have been identified as potential pharmacodynamic biomarkers and others are in the pipeline after preclinical validation. MRI and ^18^F-fluorodeoxyglucose PET can be considered useful supportive diagnostic tools for the differentiation of other HD-like syndromes.

**Summary:**

New trials in HD have the primary goal to lower mutant huntingtin (mHTT) protein levels in the brain in order to reduce or alter the progression of the disease. MR and PET molecular imaging markers have been developed as tools to monitor disease progression and to evaluate treatment outcomes of disease-modifying trials in HD. These markers could be used alone or in combination for detecting structural and pharmacodynamic changes potentially associated with the lowering of mHTT.

## Introduction

Chorea, from the Greek word for dance, is a rapid and involuntary movement affecting randomly different parts of the body. Chorea has short duration and an irregular pattern. The phenomenology is similar regardless of etiology, and therefore, clinical diagnosis is often challenging [1]. Chorea can be part of either a familial or a sporadic neurodegenerative disease. Huntington’s disease (HD) is the most common cause of chorea associated with a familial neurodegenerative disorder. After the discovery of the causative mutation in 1993 [2], considerable effort has been devoted to understand the underlying pathophysiology and to find disease-modifying treatments. Initially, caudate atrophy was the primary supportive finding for HD diagnosis. Over the years, various neuroimaging modalities have been proposed as HD biomarkers. An ongoing huntingtin lowering trial (IONIS-HTT_Rx_) illustrates the need for validated biomarkers for HD. In this review, we describe the existing structural imaging data for HD and HD phenocopies and provide a summary of promising and novel functional neuroimaging tools. Our aim is to illustrate the potential utility of different imaging modalities in clinical investigations of chorea and in clinical trials for HD.

### Huntington’s Disease

HD is caused by pathological CAG expansion in huntingtin (*htt*). Healthy subjects usually carry 7–12 CAG repeats while the range of intermediate alleles is 27–35 CAG repeats. Penetrance is incomplete for 35–39 CAG repeats and becomes complete with a larger number [3]. The nucleotide expansion size correlates inversely with age of onset and explains up to 60% of variability in age of onset [3]. CAG age product (CAP) is a useful index, applied in longitudinal studies, to estimate the time to HD phenoconversion [4]. In the majority of cases, the onset occurs during adulthood, whereas manifestations before or at age 20 defines the juvenile form (JHD). The unequivocal presence of motor signs defines a diagnosis of HD [3]. Chorea is the most common motor and characteristic feature of HD but other hyperkinetic manifestations occur in HD (dystonia, myoclonus, and vocalizations). Several voluntary and eye movements are also affected resulting in poor coordination, parkinsonism, motor impersistence, and loss of postural reflexes. Parkinsonism is more common in JHD and in later stages of adult-onset HD. Seizures and myoclonus occur in 35% of JHD cases [3]. Cognitive decline is progressive and usually precedes HD diagnosis; psychiatric/behavioral features are variable and include depression, anxiety, apathy, lack of insight, and disinhibition. The course of disease is relentlessly progressive.

### Huntington’s Disease-Like Syndromes

An etiological diagnosis is achieved only in a small fraction (2–3%) of all chorea cases tested negative for HD [5••, 6•]. Most of these Huntington’s disease-like syndromes (HDLs), also called HD phenocopies, are associated with adult-onset. Acquired cases of chorea are beyond the scope of this review and the reader is referred to other articles [7].

Expansions in the *C9orf72* gene were found to be the most common HDL (frequency 2%) in an UK cohort [6•]. Intronic hexanucleotide repeat expansions in *C9orf72* are the most common cause of familial amyotrophic lateral sclerosis (ALS) and frontotemporal dementia (FTD), two disease conditions that are often co-morbid [6•, 8]. This mutation is also associated with predominant and variable motor phenotypes (parkinsonism, hyperkinesias, and ataxia) even within the same family [9].

Mutations in prion protein gene (*PRNP*) cause inherited prion diseases (IPD) which represent up to 15% of all prion diseases. IPDs are adult-onset and fatal conditions presenting with rapidly progressive dementia, movement disorders (commonly ataxia and myoclonus) and psychiatric symptoms. Familial Creutzfeldt-Jakob disease (fCJD) is the most frequent IPD followed by Gertsmann-Sträussler-Scheinker syndrome (GSS) [10]. A Swedish family affected by a HD phenocopy was reported in 1998 [11]; despite being called Huntington’s disease-like 1 (HDL1), not all the affected subjects had chorea. Its underlying mutation is an insertion of extra 8-OPRI into the *PRNP* gene [12].

Huntington’s disease-like 2 (HDL2) affects patients of African ancestry and is caused by CTG/CAG repeat expansions in the Junctophilin-3 gene (*JPH3*) [13]. In the USA, HDL2 constitute 1% of all HDL case [14]. Clinical characteristics overlap substantially with HD and the diagnosis relies on genetic testing.

Spinocerebellar ataxia type 17 (SCA17), originally described in Japan, is caused by a pathological CAG expansion is in the TATA-box-binding protein gene (*TBP*) [15]. Age of onset is variable and correlates poorly with size of CAG repeats. The main features are ataxia, dementia, involuntary movements (chorea and dystonia), and psychiatric symptoms; parkinsonism and seizures occur in some cases [16]. Screening of cohorts with different ethnicity have established that SCA17 is the second most common HDL in Western populations [5••, 17].

Benign hereditary chorea (BHC) is an early-onset condition presenting with movement disorders, developmental delay, pyramidal symptoms, and non-neurological manifestations such as lung disease and hypothyroidism [18]. The disease is caused by mutations in the Thyroid transcription factor-1 gene (*TIFT-1*) with brain-lung-thyroid syndrome as the most common phenotype, but isolated chorea has been described as generalized, non-progressive, and paradoxically attenuated by levodopa. Other movement disorders include ataxia, dystonia, tremor, and myoclonus [19, 20]. Mutations in the adenylate cyclase type 5 gene (*ADCY5*) are associated with familial and sporadic BHC with early-onset and non-progressive symptoms. Hyperkinesia with marked episodic exacerbations particularly at night and facial twitches are other striking features; delay of motor milestones occurs in the most severe presentations [21, 22].

The core neuroacanthocytosis syndromes include chorea-acanthocytosis (ChAc) and McLeod syndrome (MLS). Both are adult-onset and common features are progressive huntingtonism, vocalizations, and feeding dystonia. Parkinsonism is common in later disease stages. Cognitive decline, pronounced psychiatric symptoms, epilepsy, neuromuscular manifestations such as myopathy and polyneuropathy, and acanthocytosis are also common [23]. Sudden truncal flexions, head drops, and buckling of the knees characterize ChAc [24]. MLS is rarer than ChAc with 150 known cases [25].

Neuroferritinopathy and aceruloplasminemia are two of the diseases included in the group called neurodegeneration with brain iron accumulation (NBIA). Both are adult-onset and associated with heterozygous respectively biallelic mutations in ferritin light polypeptide (*FTL*) and *C gene* [26]. Chorea is the predominant feature in neuroferritinopathy; chorea, ataxia, diabetes, and retinopathy define the core symptoms for aceruloplasminemia [26, 27].

Chorea can be one feature of mixed hereditary ataxias, mainly spinocerebellar ataxia types 1, 2, and 3 (SCA1, SCA2, SCA3), dentatorubral-pallidoluysian atrophy (DRPLA), Friedreich ataxia, ataxia telangiectasia, and ataxias with oculomotor apraxia. Chorea is common in paroxysmal dyskinesias and occurs in connection with mitochondrial disorders particularly mitochondrial encephalomyopathy, lactic acidosis, and stroke-like episodes (MELAS) and Leigh syndrome and in neurometabolic conditions (e.g., Lesch-Nyhan syndrome, GLUT1 deficiency, and phenylketonuria) [28].

## Magnetic Resonance Imaging

### Structural Volumetric MRI in HD

Striatal atrophy has been the most consistent, sensitive, and robust finding in HD in several observational studies [29, 30]. The largest of them, PREDICT and TRACK HD, have confirmed that this atrophy preceded phenoconversion by several years and progresses into the manifest disease phase [31••, 32••, 33••, 34]. For this reason, striatal atrophy has been proposed as a biomarker for future clinical trials [35]. Striatal atrophy is the result of progressive neuronal death and gliosis with particular vulnerability for medium spiny neurons (MSN) following caudo-rostral and dorso-ventral directions [36]. In addition, PREDICT and TRACK HD studies have revealed that later during the premanifest phase, variable cortical and white matter (corpus callosum, the posterior tracts, and the white matter surrounding the striatum) atrophy becomes evident [37]. On the other hand, atrophy is less pronounced in globus pallidus, thalamus, and hippocampus. Volumes of striatum and gray matter at baseline are predictors of HD diagnosis and loss of striatal volume and degree of cortical atrophy correlate with CAG repeat size [38, 39]. In patients with manifest disease, the pattern of cortical atrophy was found to be topographically selective [40]. Estimates of atrophy correlate with functional, motor, and cognitive decline in different structures [31••]. Recently, a clinical trial demonstrated that creatine slowed down the progression rate of striatal atrophy, but the examined clinical parameters did not change [41], suggesting that there are other factors other than striatal neurodegeneration that are linked to the complex symptomatology.

### Structural MRI in Huntington’s Disease-Like Syndromes

Neuroimaging data for *C9orf72* with HD-like phenotype are available only for 4 patients and compatible with generalized atrophy [6, 42, 43]. Several studies on *C9orf72* FTD have shown symmetric cortical atrophy in frontal, temporal and parietal lobes, thalamus, cerebellum, and corticospinal tracts [8, 44]. Similar findings were obtained in a longitudinal study on mutation carriers with ALS, FTD, and combined ALS-FTD [45, 46]. Atrophy in thalamus is a recurrent finding and may be useful to differentiate from sporadic FTD [47, 48].

In HDL1/inherited prion diseases, generalized brain and cerebellar atrophy occurs in both 6-OPRI and 8-OPRI but these findings are not specific [49–51]. This is in contrast to sporadic and variant CJD for which neuroimaging can be a supportive tool for the diagnosis [52].

In HDL 2, there is an inverse correlation between the nucleotide expansion size with age of onset and caudate atrophy. Striatal and generalized atrophy were evident in 19 out of 20 patients [14] and radiologically indistinguishable from HD [13].

In the SCA17, salient neuroimaging features include aspecific cerebellar and cerebral atrophy [16] and in one study hyperintensities in the putamen have been described [53].

In benign hereditary chorea, neuroimaging features are in the majority of cases normal but variable and non-specific abnormalities that include cerebral and cerebellar atrophy, pituitary cysts, and hyperintensities in the pallidum and vermis on T2-weigthed images have been described for the brain-lung-thyroid syndrome [54, 55]. Surprisingly, structural neuroimaging is normal in ADCY5-related dyskinesias [21, 56].

Similar to HD, in neuroacanthocytosis syndromes, structural neuroimaging demonstrates both caudate atrophy with dilatation of the anterior horns and variable cortical atrophy [57, 58]. MRI commonly shows T2-weighted signal increase in the striatum along with hippocampal sclerosis and atrophy [59, 60]. Cerebellar atrophy is a rare feature [61] and basal ganglia iron accumulation has also been reported [62]. In McLeod syndrome, striatal atrophy is progressive and inversely correlated with disease duration [63, 64]; less frequent are white matter hyperintensities [63]. Neuroimaging can be normal in asymptomatic mutation carriers [65].

In neuroferritinopathy and aceruloplasminemia, both brain MRI gradient echo (T2*) and fast spin echo modalities reveal abnormalities highly suggestive of neuroferritinopathy [66]. Progressive iron accumulation has been described in all affected and even in asymptomatic mutation *FTL* carriers [66]. This deposition is visualized on T2 images as hypointensity in the red nucleus, substantia nigra, putamen, pallidus, thalamus, and cerebral cortex. Later on, tissue damage leads to cavitations in the caudate and putamen [66]. In aceruloplasminemia iron accumulation is evident in the thalamus and caudate nucleus [67]. An overview of the main findings is presented in Table 1.

**Table 1:** Summary of morphological abnormalities using MRI for the main forms of familial huntingtonism. Caudate atrophy is similar in HD, HDL2, and neuroacanthocytosis syndromes

Disease	Pattern of inheritance	Main findings	References
Huntington’s disease (HD)	AD	Progressive striatal atrophy antedating phenoconversion for years Variable degree of atrophy in cortical regions and white matter evident in the premanifest phase	Paulsen et al. [34] Aylward et al. [33••] Tabrizi et al. [32••]
*C9orf72*-related diseases	AD	Generalized atrophy seen *C9orf72* huntingtonism but data is limited (6 patients)	Mahoney et al. [8], Hensman et al. [6•]
Huntington’s disease-like 1 (HDL1)/inherited prion diseases (IPD)	AD	Generalized cerebral and cerebellar atrophy	Xiang et al. [11] Mead et al. [51]
Huntington’s disease-like 2 (HDL2)	AD	Striatal atrophy in all reported cases, variable degree of cortical atrophy	Anderson et al. [14]
Spinocerebellar ataxia type 17 (SCA17)	AD	Generalized cerebral and cerebellar atrophy	Toyoshima and Takahashi [16]
Benign hereditary chorea (BHC)	AD	In general normal; cerebral and cerebellar atrophy and hypoplastic pallidum can occur in BLTa	Adam and Jankovic [54]
ADCY5-related dyskinesia	AD	Normal findings	Mencacci et al. [56]
Neuroacanthocytosis			
Chorea-acanthocytosis (ChoAc)	AR	ChoAch: Vast majority displays striatal atrophy, striatal hyperintensities are common Striatal atrophy indistinguishable in both ChoAc and MLS, more prominent in the caudate head Progressive striatal atrophy demonstrated in MLS	Gradstein et al. [57] Walterfang et al. [58] Valko et al. [64]
McLeod syndrome (MLS)	XLR		
NBIA			
Neuroferritinopathy	AD	T2: Hypondense BG, cortex, red nucleus, and substantia nigra. Cavitations in caudate and putamen	McNeill et al. [66]
Aceruloplasminemia	AR	Iron accumulation in thalamus and caudate, absence of cavitations	Miyajima [68]

*AD* autosomal dominant, *AR* autosomal recessive, *BG* basal ganglia, *BLT* brain-lung-thyroid syndrome, *XLR* X-linked recessive

### Diffusion Imaging

Diffusion imaging techniques measures motion features of water molecules in different tissues (e.g., white/gray matter) [69]. The most relevant clinical application is diffusion-weighted imaging (DWI) that allows an early detection of ischemic lesions, a more specific delineation of the infarction core volume in stroke patients [70] and tissue characterization of brain tumors [71]. Another informative diffusion-based MR technique is diffusion tensor imaging (DTI) that allows a description of brain tissue microstructures measuring its integrity and white matter abnormalities. This is performed by examining the magnitude of anisotropic diffusion of water molecules assuming a Gaussian distribution of diffusion patterns [72]. Normal white matter fibers show an anisotropic (dependent on direction) diffusivity of water molecules due to the linear structure of axons. Among the measures obtainable from DTI analysis, fractional anisotropy (FA), and mean diffusivity (MD) are the most commonly uses. FA and MD respectively reflect the degree of anisotropy and diffusivity of water molecules at voxel level knowing that both change following microstructural re-arrangement. Changes in FA are conventionally associated with changes of microstructural coherence that might follow pathological processes such loss or gain of tissue organization (axonal density), cellular integrity (e.g., neurodegeneration), and myelination [69]. MD changes are associated with impairment of gray matters microstructure and integrity.

Convincing evidences and description of disease specific DTI patterns are already established in different neurological diseases such as amyotrophic lateral sclerosis [73], Parkinson’s disease [74], multiple sclerosis [75], Alzheimer’s disease [76], and epilepsy [77].

There are HD-specific DTI patterns that potentially may serve as a surrogate biomarker [35]. On the other hand, different cross-sectional DTI studies in HD highlighted some intrinsic methodological limitations (e.g., small-sample size and varying DTI-based imaging protocols) [78]. Studies that were comparable in design (DTI methods and metrics, regions of interest) suggested a disease-specific pattern characterized by increased FA in the striatum and globus pallidus in manifest HD mutation carriers as compared to control subjects [79–81]. Another consistent pattern has been found in the corpus callosum where a reduction of FA has been described years before the motor symptom onset [82, 83]. An increase of MD within the basal ganglia, corpus callosum, and thalamus has consistently been described also in premanifest HD mutation carriers with high CAG-associated disease burden score [80, 84, 85]. An unexpected increase of FA in the striatum might reflect a coherent breakdown of different gray and white matter constituents (e.g., microstructure simplification, cytoskeleton degeneration, myelin breakdown, loss of cortical axons) [79, 86].

Most of the recent cross-sectional studies in the field were able to show early differences in the premanifest HD mutation group by using multimodal approaches (e.g., gray matters metrics) and multivariate statistical models [84, 87]. The study from the PREDICT-HD Investigators and Coordinators of the Huntington Study Group was able to describe a central-to-peripheral and posterior-to-anterior atrophy patterns that spreads from the deep gray matter to deep white matter. Diffusion patterns were characterized by an early increase of MD and FA measurements in the basal ganglia only in premanifest HD mutation carriers with higher CAG-induced disease burden [84].

It is important to notice that diffusion-based MRI techniques are prone to noise due to different factors such as image ghosting, geometric distortions, susceptibility, and movement artifacts. These factors might lead to an increase of within-subject variability and limit the possibility to use it as longitudinal biomarker to track the disease progression. Test-retest studies have shown low levels of within-subject variability in both early HD patients and healthy controls [88]. However, lower reliabilities of the test-retest for diffusion estimates were detected in the early HD group in cerebellar and brainstem sub-regions and of some gray matters subcortical regions. Accordingly, longitudinal studies were only able to describe specific white matter changes after 18 months in symptomatic HD mutation carriers with a decreased FA in the corpus callosum and middle cingulum [35, 89]. Evidences for longitudinal differences in the diffusion profile for premanifest HD mutation carriers are weaker [90] and available only for premanifest HD mutation carriers with higher disease burden [91]. At the moment, it is difficult to identify a valid and reliable DTI-based MRI biomarker in HD. Particularly difficult is the interpretation of outcome measures in pathological conditions where diffusivity-based measures are overestimated or underestimated by the presence of changes in cellularity, axonal injury, inflammation, and vasogenic edema [92].

## Functional Imaging

### Resting-State Functional MRI

Functional MRI (fMRI)-associated techniques are based on the measurement of fluctuations in brain hemodynamics linked to neuronal activations. The measurable signal relies on blood oxygenation level-dependent (BOLD) contrast that is based by a different signal strength of brain water protons produced by the paramagnetic effects of venous blood de-oxyhemoglobin [93]. The BOLD signal is related to changes in cerebral blood flow, blood volume, and tissue oxygen consumption that typically occurs after neuronal activation [94, 95]. Signal brain maps are thereafter re-constructed indicating the activated brain sub-regions that are modulated by given cognitive or motor tasks. As opposed to paradigm- or task-based fMRI, resting-state fMRI (rs-fMRI) experiments are performed in a stimulus free environment (i.e., at rest). The principle of rs-fMRI relies on the detection and analysis of spontaneous BOLD signal alterations that are co-activating under passive moments [96]. Anatomically and functionally connected brain regions that demonstrate temporal coherence of those spontaneous fluctuations form a set of defined brain networks: default mode, fronto-parietal, attention, visual, and salience networks [97]. The description of alterations of this “intrinsic neuronal coherence” in physiological and pathological conditions, including neurodegeneration, has provided insights into the functional organization of the brain [98, 99]. Different well-designed rs-fMRI studies show consistently a decreased intrinsic connectivity pattern across all clinical stages of HD mutation carriers [100]. The earliest reductions were observed in motor, visual, and dorsal attention networks in premanifest HD mutation carriers as compared to controls followed by a more widespread distribution to subcortical gray matters, including the so called default mode network and occipital areas at early manifest disease stage [101, 102]. Interestingly, implementation of graph theory analysis showed that premanifest HD mutation carriers have a more simplified and non-efficient network structure in the proximity of the diagnosis [103]. However, in a recent cohort of HD gene mutation carriers far from the predicted clinical onset of HD, a comprehensive study assessing sensory-motor brain structures with DTI and rs-fMRI, brain function with neurophysiological examinations, and clinical measures was unable to detect significant differences as compared to healthy controls [104].

Based on rs-fMRI findings, different authors described pattern of increased connectivity in different regions as a part of a compensatory mechanism at early disease stage [105, 106].

In early symptomatic HD mutation carriers, connectivity changes in the supplementary motor area and cingulate cortex were associated with motor performances whereas changes in lateral prefrontal networks were associated with cognition when controlling for sub-regional atrophies within the involved nodes [106]. Another study found that in manifest HD mutation carriers the presence of atrophy spatially overlapped with the disrupted intrinsic network unless a voxel-wise correction for gray matter volume is performed [107].

The fMRI community is discussing certain limitations with regard to the definition of the resting condition (e.g., eyes open or closed, mental wandering) [108, 109], to the wide variety of analysis methods employed, to the variability of the BOLD signal [110], and to the presence of relevant confounders such as physiological non-brain BOLD signal fluctuations [111]. Despite the caveats, some of the high-level measures obtained with the graph theory-based analysis showed an adequate test-retest variability [112]. If rs-fMRI techniques shall be able to track the disease progression, it is important, beside the aforementioned caveats, that longitudinal studies show detectable changes in the premanifest phase. Different longitudinal studies recently failed to detect consistent connectivity changes within the premanifest period of the disease over the course of 1 to 3 years [113•, 114]. A recent cross-sectional and longitudinal study using graph theory-based analysis over 2 years (baseline, year 1, year 2) demonstrated detectable changes for global theory measures only in symptomatic HD mutation carriers which indicated a reduction in the small-world network organization of the brain [103]. Only in the premanifest HD mutation carrier cohort a reduction of hub organization was observed. Across sessions, no significant changes were detected suggesting that those measures were not reliable markers of longitudinal changes in HD [104]. According to recent opinion leaders in the field, it is difficult to identify a valid and reliable rs-fMRI biomarker in HD [100].

## Molecular Imaging

Positron emission tomography (PET) is an in vivo molecular imaging technique able to examine the distribution and concentrations of radiolabelled compounds binding to different molecular targets. Radiolabelled compounds are referred to as radioligands. In recent years ~ 400 targets have been evaluated and ~ 40 radioligands have been developed and applied to in vivo PET studies in the human brain. For research or clinical purposes, PET has been used to measure the blood flow, glucose metabolism, receptor or transporter densities, enzyme and protein densities and activities. The basic principle of the PET stands on the measurement of emitting annihilation photons that are generated from the decay of positron emitters or isotopes (^18^F, ^11^C, ^13^N, ^15^O). Positrons emitted in matter lose most of their kinetic energy until an interaction by annihilation with an electron of the surrounding matter (i.e., brain parenchyma) occurs. A PET radioligand is administered intravenously into the blood circulation of the living subject in a condition that can be defined at rest. A dynamic PET study consists in a continuous measurement of the emitted radioactivity (coincidences) in a period of time that varies among different radioligands (commonly 60–90 min) which mirrors the pharmacokinetic behavior of the given molecule in a targeted tissue or volume of interest. Changes in radioactivity concentration in the brain and in the blood are then the objective of quantification to generate physiologically meaningful objective parameters (outcome measures) able to describe different metrics of the biological targets. The biochemical fate of a radioligand and the relative tissue radioactivity concentration are influenced by several factors such as plasma protein binding, permeability of the blood brain barrier, tissue blood flow, rate of the extraction of the tracer from the capillary, binding association and dissociation rates, non-specific tissue binding, and by the presence of radiolabelled metabolites. In the current practice, mathematical models are employed to describe the precise relationship between the in vivo radioligand behavior and a set of parameters of interest in the target tissue. In recent years, efforts are made in order to improve the quantification of relevant biological parameters in pathological situation where structural changes and volume loss are expected. Particularly promising is the combined use of high-resolution MRI images and algorithms for correction of partial volume effects that might enable a better integration of structural and functional information. Different molecular targets may potentially describe the evolution of the underlining neuronal or synaptic loss in relation to the progressive and dynamic changes that are observed during the development of HD pathology in the brain. Therefore, many experts and pharma companies view PET imaging probes as potential surrogate pharmacodynamic biomarkers for HD.

### The Role of [^18^F]FDG in HD and HD-Like Syndromes

Different PET imaging studies investigating the glucose metabolism in HD mutation carriers were able to define subcortical and cortical metabolic patterns across the entire disease spectrum of the neurodegenerative process with a progressive reduction of subcortical and cortical glucose metabolism [115–117]. The reduction in striatal metabolism is an early feature that can be observed in the premanifest phase of the disease (before the motor onset of the disease). Noteworthy, the striatal reduction of glucose uptake in premanifest HDGECs preceded the beginning of neuronal loss [117, 118]. Longitudinal analysis of premanifest HDGCs using spatial covariance image approaches led to the description of a HD-metabolic progression pattern that consisted in progressive subcortical and cortical hypometabolism in the striatum, thalamus, insula, posterior cingulate gyrus, and prefrontal and occipital cortex associated to a relative hypermetabolism in the cerebellum and pons [119••]. The progressive cortical hypometabolism was correlated with the progression of the cognitive deficits [120]. Unfortunately, no specific patterns were detected for the progression of the psychiatric features of the disease. The utility in the clinical practice of [^18^F]FDG-PET is limited to rare occasions in which a suspected or confirmed HDGECs present an unclear motor onset. There are also studies that suggested a possible application of [^18^F]FDG-PET imaging as a tool to predict the time of symptomatic conversion [121].

The [^18^F]FDG-PET metabolic pattern in other non-HD neurodegenerative and acquired syndromes were recently reviewed by Ehlric and Walker [7]. Metabolic patterns that essentially mimic those observed in HD (a predominant striatal hypometabolism) have been described for different conditions such as in neurochoreacanthocitosis syndromes [60, 122], SCA17 [123], SCA19 [124], and Wilson’s disease [125]. According to the literature, the pattern reflects the neurodegenerative nature of those conditions. Of interest, [^18^F]FDG-PET imaging studies performed in acquired/non-degenerative forms of chorea such in the antiphospholipid syndrome [126], polycythemia vera [127], and Sydenham’s chorea [128] showed an increased striatal metabolism.

### The Role of Dopaminergic Imaging Markers in HD

Since the major pathological features in HD were traditionally related to the loss of MSNs in the striatum, many PET studies have been focused on post-synaptic dopaminergic receptors. Reduction of dopamine D_1_ and D_2_ receptor availability were reported both in manifest and premanifest HDGECs. Multi receptor PET studies examining D1 receptors with [^11^C]SCH23390 and D_2_ receptors with [^11^C]raclopride showed similar impairments of the two receptor systems in manifest and premanifest HDGECs [129–131]. Different PET studies performed with [^11^C]raclopride showed approximately 40–60% loss of D_2_ receptors in the striatum of manifest HDGECs and approximately 10–50% reduction in heterogeneous and small groups of premanifest HDGECs as compared to healthy control subjects [116, 132, 133]. Conflicting results have also been obtained when dopamine D_2_ receptors availability was measured in extra-striatal areas [134, 135]. The variability, between studies, regarding the reduction of D_2/3_ receptors found in premanifest HDGECs might represent the consequence of a broad range of CAG repeats and the burden of pathology among the HDGECs examined. In vivo PET studies with presynaptic markers have shown reduction of striatal VMAT2 binding have been reported in manifest HDGECs with the radioligand [^11^C]dihydrotetrabenazine with an estimated reduction of 25% in the akinetic-rigid motor syb-type [136]. In addition, in a small group of manifest HDGECs, 50% reduction of DAT binding was described with [^11^C]beta-CIT-PET [129].

### The Role of Phosphodiesterase 10A Imaging in HD

Among many interesting striatal targets, the intracellular phosphodiesterase 10A (PDE10A) enzyme has recently gained attention for the key role in the regulation of striatal signaling and for the presence of suitable pharmaceutical compounds able to modulate it [137, 138]. PDE10A is highly expressed in MSNs of the striatum, at the confluence of the cortico-striatal glutamatergic and the midbrain dopaminergic pathways, where it encodes the hydrolysis of both cyclic adenosine monophosphate (cAMP) and cyclic guanosine monophosphate (cGMP) [139]. These second messengers are important regulators of molecular signaling in the cortico-striato-thalamic circuit. It has been also reported that PDE10A inhibition improves cortico-basal function in Huntington’s disease model with detectable changes with a PDE10A PET radioligand [140••].

**Fig. 1:**
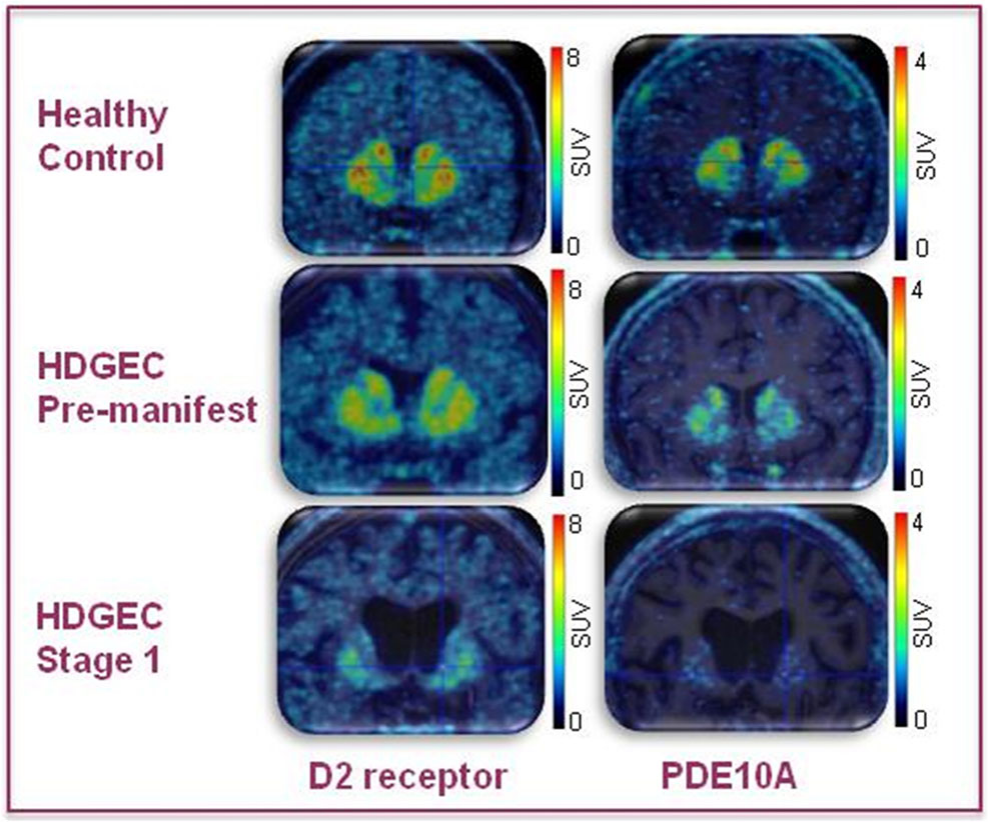
Representative standardized uptake values (SUV) PET images (D2 receptors and PDE10A enzyme) depicting coronal brain section at the level of basal ganglia for HD mutation carriers at different stages of the disease overlaid on top of individual MRI image

Several studies have already been performed in manifest and premanifest HDGECs with the aim of examining the PDE10A enzyme [141•]. All PET imaging studies investigating the availability of PDE10A in HDGECs suggested a specific alteration of this protein along with the neurodegenerative process. There are several radioligands currently validated and used in vivo in clinical PET studies: [^18^F]JNJ42259152, [^18^F]MNI-659, [^11^C]IMA107, and [^11^C]Lu AE92686. The work of Ahmad et al. [142] was the first study showing a dramatic reduction (after PVE correction) of the enzyme in five manifest HDGECs compared to 11 healthy controls that were not age-matched (70% in the caudate and 65% in the putamen). In the first cross-sectional study employing a fully validated PDE10 radioligand [^18^F]MNI-659, Russell et al. showed an average reduction of PDE10A availability of 50% in three premanifest and eight manifest HDGECs compared to age-matched healthy controls [143]. The same group has recently published a longitudinal study in which eight HDGECs (six manifest and two premanifest) underwent two [^18^F]MNI-659 PET measurements 1 year apart. On average, the estimates of annual PDE10A loss were 16.6% in the caudate, 6.9% in the putamen, and 5.8% in the globus pallidus. The outcome measures of this study were not corrected for PVE [144]. More recently, using [^11^C]IMA107 PET, a group of 12 early premanifest HDGECs (approximately 25 years before the predicted onset) showed respectively 25 and 33% reductions in the striatum and in the globus pallidus compared with age-matched healthy controls. At that “stage” in HDGECs, no evident volumetric changes were detected in the gray and white matter structures as result of a morphometric analysis [145••]. Currently our group, in collaboration with the CHDI foundation, has completed a cross-sectional and longitudinal PET study (ClinicalTrials Identifier: NCT02061722) in a large cohort of HD mutation carriers with the intention of measuring PDE10A in relation to D_2_ receptors across all different disease stages bearing in mind in the study design the necessity to develop a pharmacodynamic biomarker. Preliminary analysis of the results suggests a progressive decrease of PDE10A binding across all stages that goes beyond the D_2_ receptor and striatal volume loss. The data for this study has been already presented to international conferences [146, 147] (Fig. 1).

### Other PET Molecular Imaging Markers

In humans, other striatal markers, such as adenosine-1, GABA-A, and opioid receptors, have been examined with PET and found to be decreased in HD subjects [148]. The accumulation of mutant huntingtin protein (mHTT) is associated to downregulation of several molecular imaging markers that are expressed not only in the basal ganglia but also in cortical regions. PET imaging studies have demonstrated loss of cannabinoid-1 receptors in animal models of HD and in manifest HD patients [149–151]. Further studies across different stages of the disease are needed to examine whether these targets can be used as pharmacodynamic biomarkers for clinical trials in HD aimed at lowering mHTT.

## Conclusion

Major efforts are currently underway to identify treatment strategies that could slow down or stop the natural course of the neurodegenerative process at different levels (e.g., delivery of neurotrophic factors, activation of neuronal stem cells, autophagy, and mitochondrial function normalization). Different approaches are in the pipeline at different stages of preclinical or clinical development but currently antisense oligonucleotides (ASOs) and other RNAi-based reagents are the most promising strategies to suppress mHTT message and protein [152]. The antisense oligonucleotides delivered intrathecally has already passed the phase II trials. The ideal biomarker should be able to either measure directly the mHTT strains in the brain or detect pharmacodynamic changes associated to mHTT load. The accumulation and the role of the different mHTT strains is still unclear; therefore, a wider approach with different dedicated molecular target is mandatory at this stage. In the list of imaging biomarker presented in this review striatal volume changes and PDE10A imaging have demonstrated the capability to follow and match the HD pathological process in the basal ganglia. Currently, efforts are made to develop and validate HTT-lowering pharmacodynamic biomarkers that are linked with the amount of accumulated of mHTT in the brain. Finally, efforts are in place to enter in the clinical development a PET radioligand that targets mHTT with assessment in healthy volunteers followed by a cross-sectional study in HD mutation carriers at different stages [153].
